# Identification and Characterization of the Stability of Hydrophobic Cyclolinopeptides From Flaxseed Oil

**DOI:** 10.3389/fnut.2022.903611

**Published:** 2022-06-23

**Authors:** Adnan Fojnica, Hans-Jörg Leis, Michael Murkovic

**Affiliations:** ^1^Institute of Biochemistry, Graz University of Technology, Graz, Austria; ^2^Department of Pediatrics and Adolescent Medicine, Medical University of Graz, Graz, Austria

**Keywords:** flaxseed oil, cyclolinopeptides, characterization, stability, Orbitrap MS-RP HPLC

## Abstract

Flaxseed (linseed) is a cultivar of the spring flowering annual plant flax (*Linum usitatissimum*) from the Linaceae family. Derivatives of this plant are widely used as food and as health products. In recent years, cyclic peptides isolated from flaxseed and flaxseed oil, better known as cyclolinopeptides (CLPs), have attracted the attention of the scientific community due to their roles in the inhibition of osteoclast differentiation or their antimalarial, immunosuppressive, and antitumor activities, as well as their prospects in nanotechnology and in the biomedical sector. This study describes the detection, identification, and measurement of CLPs in samples obtained from nine different flaxseed oil manufacturers. For the first time, Q Exactive Hybrid Quadrupole-Orbitrap Mass Spectrometer was used for CLP identification together with RP-HPLC. The routine analyses were performed using RP chromatography, measuring the absorption spectra and fluorescence detection for identifying tryptophan-containing peptides using the native fluorescence of tryptophan. In addition, existing protocols used for CLP extraction were optimized and improved in a fast and cost-efficient way. For the first time, 12 CLPs were separated using methanol/water as the eluent with RP-HPLC. Finally, the stability and degradation of individual CLPs in the respective flaxseed oil were examined over a period of 60 days at different temperatures. The higher temperature was chosen since this might reflect the cooking practices, as flaxseed oil is not used for high-temperature cooking. Using HPLC–MS, 15 CLPs were identified in total in the different flaxseed oils. The characterization of the peptides *via* HPLC–MS highlighted two types of CLP profiles with a substantial variation in the concentration and composition of CLPs per manufacturer, probably related to the plant cultivar. Among the observed CLPs, CLP-O, CLP-N, and CLP-B were the least stable, while CLP-C and CLP-A were the most stable peptides. However, it is important to highlight the gradual degradation of most of the examined CLPs over time, even at room temperature.

## Introduction

Flaxseed (linseed) is a cultivar of the spring flowering annual plant flax (*Linum usitatissimum*) from the Linaceae family (1). Flax was first domesticated in approximately 8,000 B.C. in southwest Asia (Turkey, Iran, Jordan, and Syria) and is native to Algeria, Egypt, Tunisia, Greece, Italy, Spain, and Asia Minor (2). It is primarily used for direct consumption or as a salad oil and as an ingredient in traditional medicine (3). Currently, flaxseed is cultivated in more than 50 countries, including Canada, the United States, Brazil, India, China, and Ethiopia (4). Canada is the leading producer and exporter of oil-type flaxseed, producing on average 1,260,000 tons of flaxseed for oil, meal, and fiber, representing 43% of world production (5). The United States produces 224,000 tons of linseed annually (5). In Europe, the main producers of flax are the Czech Republic, Slovakia, France, Germany, Italy, Belgium, and Ireland (6). The plant is primarily cultivated to produce fibers for linen production, while plants grown for oilseed are of the same species but belong to a different cultivar (6).

Major constituents of flaxseed on average are 41% fat, 28% fiber, 20% protein, 7.7% moisture, and 3.4% ash, indicating that the percentage varies depending on the cultivar, growing conditions, and seed processing (7). Other components that are commonly found in flaxseed are cyclolinopeptides (CLPs), lignans, phenols, phytic acid, cyanogenic glycosides, trypsin inhibitor, linatine, and condensed tannins (8, 9).

Cyclolinopeptides, also called orbitides or linusorbs, represent a group of cyclic, hydrophobic peptides composed of eight (octapeptides), nine (non-apeptides), or even ten (decapeptides) amino acids, with molecular weights in the range of 950–2,300 Da (10, 11). CLPs are homomono-cyclopeptides and belong to the type VI (Caryophyllaceae) cyclopeptides (12).

The first cyclopeptide ever identified was cyclolinopeptide A, extracted in 1959 by Kaufmann and Tobschirbel (13) from the deposited sediments of crude flaxseed oil. Subsequently, each newly discovered cyclic peptide from flaxseed was named after the next letter in the alphabet. In total, 39 cyclolinopeptides were identified in flaxseed oil, roots, and seeds (10, 11), while 15 cyclolinopeptides are abundantly found in flaxseed oil, including peptides from CLP-A to CLP-O (14). Novel nomenclature was proposed by Shim et al. (10). However, the results described in this manuscript use the nomenclature commonly found in the literature with three-letter codes (8, 9, 13, 15–18). The CLPs detected in this study are shown in Table 1.

**Table 1:** CLPs in flaxseed oil extract detected using Hybrid Quadrupole-Orbitrap Mass Spectrometer-RP HPLC.

**Type**	**Sequence**	**Chemical formula (MW + H^**+**^)**	**References**	**Oils in which CLPs are detected**
CLP-A	Ile-Leu-Val-Pro-Phe-Phe-Leu-Ile	C_57_H_85_N_9_O_9_ (1,040.6543)	(13)	“AN,” “BL,” “SP,” “DM,” “ES,” “FA,” “GA,” “PB,” and “VD”
CLP-B	Met-Leu-Ile-Pro-Pro-Phe-Phe-Val-Ile	C_56_H_83_N_9_O_9_S (1,058.6107)	(8)	“BL,” SP,” “DM,” “ES,” “FA,” “PB,” and “VD”
CLP-C	Mso-Leu-Ile-Pro-Pro-Phe-Phe-Val-Ile	C_56_H_83_N_9_O_10_S (1,074.6056)	(19)	“AN,” “BL,” “SP,” “DM,” “ES,” “FA,” “GA,” “PB,” and “VD”
CLP-D	Mso-Leu-Leu-Pro-Phe-Phe-Trp-Ile	C_57_H_77_N_9_O_8_S (1,064.5638)	(19)	“AN,” “BL,” “SP,” “DM,” “ES,” “FA,” “GA,” “PB,” and “VD”
CLP-E	Mso-Leu-Val- Phe- Pro-Leu-Phe-Ile	C_51_H_77_N_8_O_9_S (977.5529)	(19)	“AN,” “BL,” “SP,” “DM,” “ES,” “FA,” “GA,” “PB,” and “VD”
CLP-F	Mso-Leu-Mso-Pro-Phe-Phe-Trp-Val	C_55_H_73_N_9_O_10_S_2_ (1,084.4995)	(15)	“AN,” “BL,” “SP,” “DM,” “ES,” “FA,” “GA,” “PB,” and “VD”
CLP-G	Mso-Leu-Mso-Pro-Phe-Phe-Trp-Ile	C_56_H_75_N_9_O_10_S_2_ (1,098.5151)	(15)	“AN,” “BL,” “SP,” “DM,” “ES,” “FA,” “GA,” “PB,” and “VD”
CLP-I	Met-Leu-Mso-Pro-Phe-Phe-Trp-Val	C_55_H_73_N_9_O_9_S_2_ (1,068.5045)	(9)	“DM” and “ES”
CLP-K	Msn-Leu-Ile-Pro-Pro-Phe-Phe-Val-Ile	C_56_H_83_N_9_O_11_S (1,090.6006)	(16)	“GA”
CLP-L	Met-Leu-Val-Phe-Pro-Leu-Phe-Ile	C_51_H_76_N_8_O_8_S (961.5580)	(16)	“SP,” “DM,” “ES,” “FA,” and “VD”
CLP-M	Met-Leu-Leu-Pro-Phe-Phe-Trp-Ile	C_57_H_83_N_9_O_8_S (1,048.5689)	(16)	“BL,” “SP,” “DM,” “ES,” “FA,” “PB,” and “VD”
CLP-N	MET-Leu-Met-Pro-Phe-Phe-Trp-Val	C_55_H_73_N_9_O_8_S_2_ (1,052.5096)	(16)	“BL,” “SP,” “DM,” “ES,” “FA,” and “VD”
CLP-O	Met-Leu-Met-Pro-Phe-Phe-Trp-Ile	C_56_H_75_N_9_O_8_S_2_ (1,066.5253)	(16)	“BL,” “SP,” “DM,” “ES,” “FA,” and “VD”
CLP-P	Met-Leu-Mso-Pro-Phe-Phe-Trp-Ile	C_56_H_75_N_9_O_9_S_2_ (1,082.5202)	(16)	“BL,” “SP,” “DM,” “ES,” “FA,” “PB,” and “VD”
CLP-T	Mso-Leu-Met-Pro-Phe-Phe-Trp-Val	C_55_H_73_N_9_O_9_S_2_ (1,068.5045)	(17)	“BL,” “SP,” “DM,” “ES,” “FA,” and “VD”

^*^Ile, isoleucine; Leu, leucine; Val, valine; Pro, proline; Phe, phenylalanine; Trp, tryptophan; Met, methionine; Msn, methionine sulfone; Mso, methionine sulfoxide.

The natural function CLPs have in plants is still unknown. However, an increasing number of biological activities of hydrophobic CLPs have been examined, and diverse effects of CLPs have been identified, such as immunosuppressive, antimalarial, cancer-inhibiting, tumor-inhibiting, and osteoclast differentiation inhibiting effects (8, 9, 20–22). As each CLP has a unique biological activity, the potential treatment with flaxseed oil will depend on the presence of the respective peptide and its concentration in the oil.

To better understand CLPs and optimize their application, the development of an efficient extraction method would be a prerequisite step. Due to the complexity and structural similarity, protocols in the past had difficulties in CLP separation and detection, where using methanol/water as eluent, only five CLPs could be identified and separated (23, 24). Due to frequent encounters with unpleasant and bitter flavors during flaxseed oil consumption, a lack of consumer acceptance of flaxseed oil is apparent. Research conducted in previous years revealed that CLPs (primarily methionine oxidized variants of CLPs) contribute to the bitterness of the oil (23, 25, 26). Additional effort should be made to examine commercially available flaxseed oils and the levels of oxidation taking place in them. Furthermore, the stability of individual CLPs in flaxseed oil at room temperature (25°C) has not been reported in the literature to our knowledge. In 2007, Brühl and his team tried to visualize the effect of storage on the development of bitter taste in flaxseed oil. For this purpose, they examined flaxseed oil over a period of 30 weeks at −18 and 22°C (23). In 2014, Lao and his group examined the forced aging of a fresh flaxseed oil sample at 68°C using the Schaal oven test (17). The most extensive work on CLP stability was performed in 2013 by Aladedunye and his group (24). They examined the stability of flaxseed oil at 65°C for 30 days, evaluated the storage of CLP extracts for 25 h and 1 month at 4°C, and finally examined CLP concentrations in flax meals stored at room temperature for up to 48 months (24).

The objective of this study was to develop an efficient, novel, rapid, and cost-efficient extraction method as a prerequisite step for a better understanding of CLPs. Additionally, we aimed to find an appropriate high-resolution HPLC column for optimal separation of the CLPs among various C18 columns. Furthermore, an examination of different flaxseed oils, commonly found on the shelves of grocery stores in Central Europe, aimed to observe different patterns of CLPs and their oxidation. The next objective was to use a Q Exactive Hybrid Quadrupole-Orbitrap Mass Spectrometer coupled with RP-HPLC for the first time to detect and identify CLPs. Finally, the behaviors of 11 individual CLPs was examined over a period of 60 days at room temperature, and the effects of higher temperatures on the CLPs were investigated.

## Materials and Methods

### Flaxseed Oil Samples and Chemicals

Abbreviations were used for flaxseed oil samples, and only the authors of this publication are familiar with the exact names of manufacturers. Cold-pressed flaxseed oil samples were acquired from the “AN” (Darmstadt, Germany), “VD” (Vienna, Austria), “ES” (Graz, Austria), “DM” (Karlsruhe, Germany), “BL” (Wels, Austria), “PB” (Spittal an der Drau, Austria), “GA” (Slovenska Bistrica, Slovenia), “SP” (Salzburg, Austria), and “FA” (Pöllau, Austria). Flaxseed oil samples were packaged in 250-ml dark bottles and stored at 4°C. All samples were freshly procured from local markets, except that flaxseed oil from the “GA” manufacturer was purchased in 2015 and was used multiple times before this experiment.

The HPLC grade deionized water, ethanol, and methanol were supplied by ChemLab Analytical (Zedelgem, Belgium).

### Extraction of Cyclolinopeptides

Cyclolinopeptides were isolated from flaxseed oil using both liquid–liquid extraction methods and sonication-assisted methanol extraction (SAME). Both methods were previously reported to be used in 2013 and 2014, and their optimized versions are reported here (17, 24). Additionally, for the first time during liquid–liquid extraction, a novel method was introduced where a simple, additional step of using a methanol-water mixture preheated at 90°C was added before the extraction. In a liquid–liquid extraction method, an aliquot of 700 μl of flaxseed oil was added to a 700 μl methanol/ethanol + water mixture, followed by vortexing for 10 min and centrifugation for 10 min at 14,000 rpm. After centrifugation, the supernatant was used for analysis. Ratios of 60:40, 70:30, and 80:20 methanol–water mixtures were tested. In addition to liquid–liquid extraction, SAME was applied, for which 1 ml of flaxseed oil was added to 1 ml of methanol, vortexed for 10 mins, and sonicated at 30°C for 1 h. The mixture was centrifuged at 14,000 rpm for 10 mins, and the supernatant containing the peptides was analyzed by RP-HPLC. Variations in methanol volume (3, 5, 7, and 10 ml) and temperature (50 and 70°C) were also investigated. As some CLPs are more prone to heat and oxidation, normal liquid–liquid extraction without a preheating step was used for all other experiments.

### CLPs Separation Using RP-HPLC

High-performance liquid chromatography was conducted using an Agilent1100 series HPLC system. This comprised a quaternary pump, a degasser, a thermostated autosampler, and a diode array detector. The samples were separated using four different columns: Grace^TM^ Vydac (5 μm; 250 × 4.6 mm; 300 Å; Hichrom, Lutterworth, United Kingdom), Gemini^TM^ C18 (3 μm; 150 × 3 mm; 110 Å; Phenomenex, Aschaffenburg, Germany), Kinetex^TM^ C18 (5 μm; 150 × 3 mm; 100 Å; Phenomenex, Aschaffenburg, Germany), and Kinetex^TM^ C18 (2.6 μm; 100 × 3 mm; 100 Å; Phenomenex, Aschaffenburg, Germany). The separation was performed using an isocratic mobile phase of 70% methanol (A), 30% water, and 5% methanol (B) at 25°C with a flow rate of 0.5 ml/min. The column compartment temperature was 25°C for all experiments. The run time was set to 15 min with an injection volume of 5 μl. The detection was performed at a wavelength of 214 nm for the determination of the peptide bonds, while 280 and 260 nm were used for tryptophan and phenylalanine detection, respectively. As the quantitation of CLP content was not the focus of this project, the injection of CLP extracts was intended to determine the presence, levels, and patterns of cyclic peptides. As the content of the CLPs is proportional to the peak height (mAU) of the detector, the response at 214 nm was used to evaluate the stability.

### CLP Patterns of Nine Different Flaxseed Oil Manufacturers

The CLP levels, presence, and pattern differ among manufacturers due to different flaxseed varieties and oil processing techniques. Each sample was analyzed separately and compared. Aliquots of 700 μl were taken from each oil. To each tube containing flaxseed oil, 700 μl of a methanol–water mixture (70:30; *v/v*) was added. The combined mixture was vortexed for 10 min and centrifuged for 10 mins at 14,000 rpm and 4°C. The peptides were found in the supernatant, while the lower phase was discarded. In addition to individual measurements of each oil using RP-HPLC, 150 μl of each CLP extract was combined in one Eppendorf tube and further analyzed by RP-HPLC coupled with a Q Exactive Hybrid Quadrupole-Orbitrap Mass Spectrometer.

### Cyclolinopeptide Detection and Identification Using Q Exactive Hybrid Quadrupole-Orbitrap Mass Spectrometer

High-resolution mass spectrometry (HRMS) spectra were recorded using a high-resolution Q Exactive Hybrid Quadrupole-Orbitrap Mass Spectrometer (Thermo Scientific) coupled with RP-HPLC. Q Exactive Orbitrap MS was used together with a Thermo Scientific Accela Open Autosampler, Thermo Scientific Accela 1,250 Pump, MayLab MistraSwitch New Generation (NG) System Solution, and Phenomenex Kinetex 2.6 μm C18 100 Å, LC 150 × 3 mm. The parameters of HPLC–MS were as follows: 45 min of stop time; scan range: 150–2,000 m/z, resolution: 140,000 (MS1) and 35,000 (MS2); positive polarity; data-dependent MS^2^ peak apex triggering (15 s); LC-flow rate: 0.5 ml/min, and an injection volume of 10 μl.

### CLP Stability in Flaxseed Oil Over a Period of 60 Days at Room Temperature and 90°C

The stability and behavior of CLPs were examined at room temperature and 90°C. In general, biochemical stability and structural rigidity are notable features of cyclic peptides, and no significant change would be expected at lower temperatures (27). Additionally, the primary structure and number of amino acids of CLPs significantly differ and impact many properties of CLPs, including biological activity. Therefore, the stability of individual CLPs was expected to vary depending on their primary structure. As flaxseed oil is not used for cooking at high temperatures (e.g., frying and roasting) but could be added during boiling, the high temperature of 90°C was chosen as the simulation for the boiling process.

For this experiment, flaxseed oil samples from the “PB” and “FA” manufacturers were used. Aliquots of 700 μl were prepared for day 1, day 5, day 10, day 20, day 40, and day 60 and heated over a defined period in an Eppendorf Thermomixer at 300 rpm. Additional time points of 30 min, 1 h, 2 h, 4 h, and 6 h were incubated at 90°C. To test the stability at 25°C (as suggested by IUPAC), the same experimental setup was used. All samples were prepared at least in duplicate.

## Results and Discussion

### CLP Extraction Methods and Efficiency of Extraction

For the purpose of examining CLP extraction methods, moderately aged flaxseed oil “PB” was used in this experiment, but the extraction methods for the “FA” sample are shown as well. There was a significant difference in CLP extraction using the liquid–liquid, hot methanol, and SAME methods, as shown in Table 2.

**Table 2:** CLP extraction methods and their efficiencies.

**CLPs**	**Methanol-water extraction (mAU)^**a**^**	**Hot methanol-water extraction (mAU)^**a**^**	**Ethanol-water extraction (mAU)^**a**^**	**SAME (mAU)^**a**^**	**Methanol-water extraction (mAU)^**b**^**	**Hot methanol-water extraction (mAU)^**b**^**
**CLP-F**	125 ± 1^a^	173± 1^b^	106 ± 2^c^	99 ± 2^d^	3 ± 0^e^	4 ± 1^e^
**CLP-G**	314 ± 1^a^	452 ± 2^b^	274 ± 4^c^	264 ± 1^d^	13 ± 1^e^	16 ± 1^f^
**CLP-C**	314 ± 1^a^	403 ± 1^b^	280 ± 1^c^	266 ± 1^d^	66 ± 2^e^	82 ± 1^f^
**CLP-D**	100 ± 1^a^	113 ± 2^b^	88 ± 1^c^	96 ± 3^d^	N.A.	N.A.
**CLP-E**	181 ± 1^a^	212 ± 1^b^	178 ± 2^c^	181± 1^d^	N.A.	N.A.
**CLP-A**	296 ± 1^a^	283 ± 2^b^	325 ± 1^c^	322 ± 1^d^	403 ± 4^e^	474 ± 1^f^
**CLP-M**	33 ± 1^a^	7± 1^b^	25 ± 1^c^	23 ± 1^d^	261 ± 1^e^	303 ± 1^f^
**CLP-T**	N.A.	N.A.	N.A.	N.A.	29 ± 1^e^	40 ± 1^f^
**CLP-P**	N.A.	N.A.	N.A.	N.A.	118 ± 1^e^	140 ± 1^f^
**CLP-N**	N.A.	N.A.	N.A.	N.A.	65 ± 1^e^	76 ± 1^f^
**CLP-B**	N.A.	N.A.	N.A.	N.A.	307 ± 2^e^	348 ± 1^f^
**CLP-O**	N.A.	N.A.	N.A.	N.A.	177 ± 4^e^	203 ± 3^f^

*Values are given as height with the standard deviation of three experiments*.

a *“PB”, sample*;

b *“FA”, sample; N.A., not available. Different letters in each line reflect a significant difference of the ANOVA test of the analyses*.

All tested methods showed good reproducibility. Overall, the most efficient method for most CLPs is the hot methanol–water extraction method. This is a simple and fast protocol reported for the first time. Five of the seven cyclolinopeptides were significantly extracted more efficiently in the hot methanol/water mixture compared to the other methods for the “PB” sample. For the “FA” oil, the extraction was improved for all CLPs, including CLP-A and CLP-M.

It is important to stress here that even though there was no momentous improvement in CLPs extraction efficiency in the examined methanol and water ratios used during liquid–liquid extraction, the 70:30 methanol ratio was the most efficient in terms of CLP levels obtained. Additionally, for SAME, sonication at different temperatures (30, 50, and 70°C) gave almost identical results. Increasing the methanol content resulted in higher extraction of triglycerides.

In 2010, Marr et al. (28) reported a 5-fold increase in CLP recovery using liquid–liquid extraction compared to silica column extraction. However, the use of silica columns for the extraction for CLP isolation is a common approach used over the years (14, 23, 29, 30). In 2013, Aladedunye et al. reported SAME to be a more efficient method compared to silica column or solvent extraction conducted in 2010 and 2011 (24, 28, 31). As the exact concentration of the CLPs is unknown in the experiments conducted here, only the UV absorption of the CLPs could be compared. This was especially useful for the very stable CLP-A. Based on CLP levels reported in their protocols, the optimized methods gave slightly better results compared to most previous protocols. However, the injection volume they used was higher than the injection volume used in our experiments.

### Comparison of the RP-HPLC Separation of Cyclolinopeptides Using Four Different Columns

**Figure 1:**
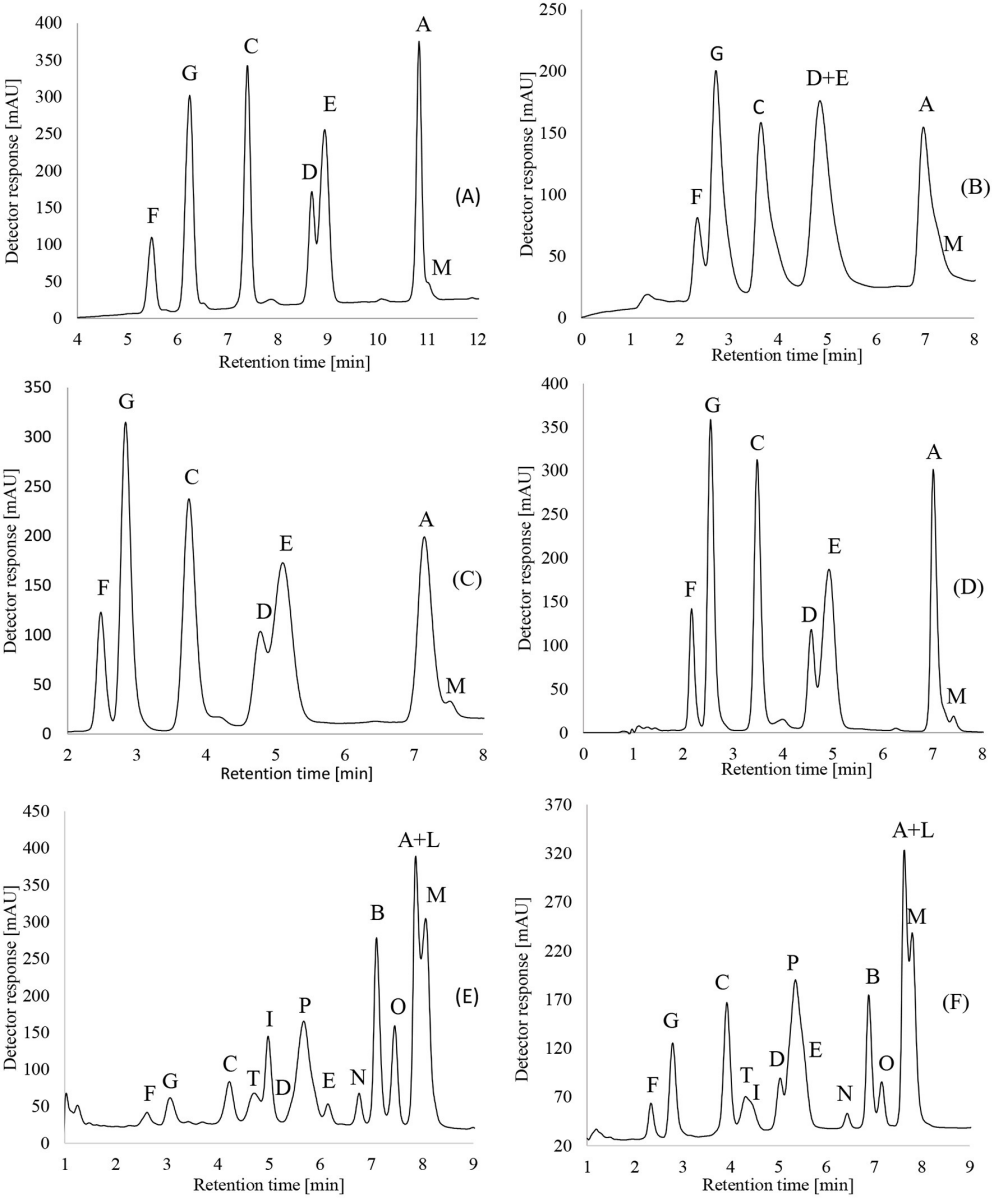
RP-HPLC separations of CLPs from a moderately aged flaxseed oil sample using **(A)** GeminiTM C18, 3 μm column, **(B)** VydacTM C18, 5 μm column, **(C)** KinetexTM C18, 5 μm column, and **(D)** KinetexTM C18, 2.6 μm column and application of KinetexTM C18, 2.6 μm column for separations of CLPs from fresh flaxseed oil sample, “DM” manufacturer **(E)** and combined flaxseed oil samples **(F)**. The following settings were used: 0.5 ml/min flow rate; methanol and water isocratic elution as described in the “Methods” section. Liquid–liquid extraction was used for the extraction.

The chromatographic performance of the two Kinetex^TM^ reversed-phase HPLC columns with different particle sizes was compared to that of reversed-phase HPLC columns from Gemini^TM^ and Vydac^TM^ to evaluate their capability for CLP mixture separation. The optimized method was used for routine analyses as well as the MS experiments. An identical gradient, flow, and temperature were used for each of the described columns as previously described in the methods, with no further need for additional optimization. The good chromatographic separation of the CLPs allowed the use of UV detection for the determination of the major CLPs. MS was only used for unambiguous identification of the cyclic peptides. For the routine analyses, UV detection (214 nm for determination of the peptide bond, 260 nm for phenylalanine residues, and 280 nm for tryptophan residues) could be used. In Figure 1, RP-HPLC separation of the CLPs is shown.

The overall retention time of the peptides was lower on the Kinetex^TM^ and Vydac^TM^ columns than onthe Gemini^TM^ column. The Gemini^TM^ and Kinetex^TM^ C18 columns with particle diameters of 3 and 2.6 μm performed better than the columns with a particle size of 5 μm (Vydac^TM^ and Kinetex^TM^), and separation was better for both the column and the CLP levels detected. Even though the Vydac^TM^ column is generally used for proteins and peptides, it seems that columns with smaller particle sizes, such as Kinetex^TM^ and Gemini^TM^ C18 columns, are more suitable for the separation of octa- and non-apeptides. Additionally, the Kinetex^TM^ C18 column with a particle diameter of 5 μm had a better separation, resulting in higher peaks of the CLPs compared to the Vydac^TM^ column.

For the “PB” sample using Kinetex^TM^ C18 columns, 7 peaks could be identified and separated, which are CLP-F, CLP-G, CLP-C, CLP-D, CLP-E, CLP-A, and CLP-M, similar to the Gemini^TM^ C18 column. Using the Vydac^TM^ C18 column, 7 peaks could also be identified. However, the separation of the pairs CLP-D and CLP-E, CLP-A, and CLP-M was not possible. The Kinetex^TM^ C18 column with a particle size of 2.6 μm had the best overall separation and was used for further experiments, including CLP identification with Orbitrap MS-RP HPLC. For the fresh sample, using a Kinetex^TM^ C18 column with a 2.6 μm particle size, up to 14 cyclolinopeptides could be detected, and 12 of them were effectively separated, CLP-F, CLP-G, CLP-C, CLP-T, CLP-I, CLP-D, CLP-P, CLP-E, CLP-N, CLP-B, CLP-O, CLP-A, CLP-L, and CLP-M. Efficient separation of CLP-A and CLP-L was not achieved.

In previous studies of chromatographic conditions for CLP separation, Brühl et al. (23) separated 5 CLPs using a LiChrospher 100 RP-18 column and methanol/water eluent. In 2012, Bo Gui et al. (14) managed to separate 6 CLPs using a ZORBAX Eclipse XDB-C18 column and acetonitrile/water eluent. In 2012, 7 cyclolinopeptides were separated using ZORBAX Eclipse XDB-C18 and Chromolith columns, again using acetonitrile-water as an eluent (29). In 2014, Lao et al. (17) separated up to 10 CLPs using a Kinetex^TM^ phenyl-hexyl column and an acetonitrile-water gradient with RP-HPLC. Using a fast and cost-efficient approach with methanol-water as the eluent, we were able to detect 14 CLPs in freshly prepared flaxseed oil (e.g., “DM” manufacturer), while separation was efficient for up to 12 CLPs using RP-HPLC. Detection and separation of CLPs were achieved within 10 min using a flow of 0.5 ml/min. Additionally, it is important to mention that some other groups managed to separate 11 and 12 CLPs using RP-HPLC. However, applying time-consuming and expensive approaches for extraction and elution, where gradients of acetonitrile-water were used, with flows of 2 and 16 ml/min (30, 32). A potential limitation of using methanol as the eluent would be an occasional drift in the baseline, which was also reported in other publications (24).

### CLP Patterns in Nine Different Flaxseed Oil Manufacturers

**Figure 2:**
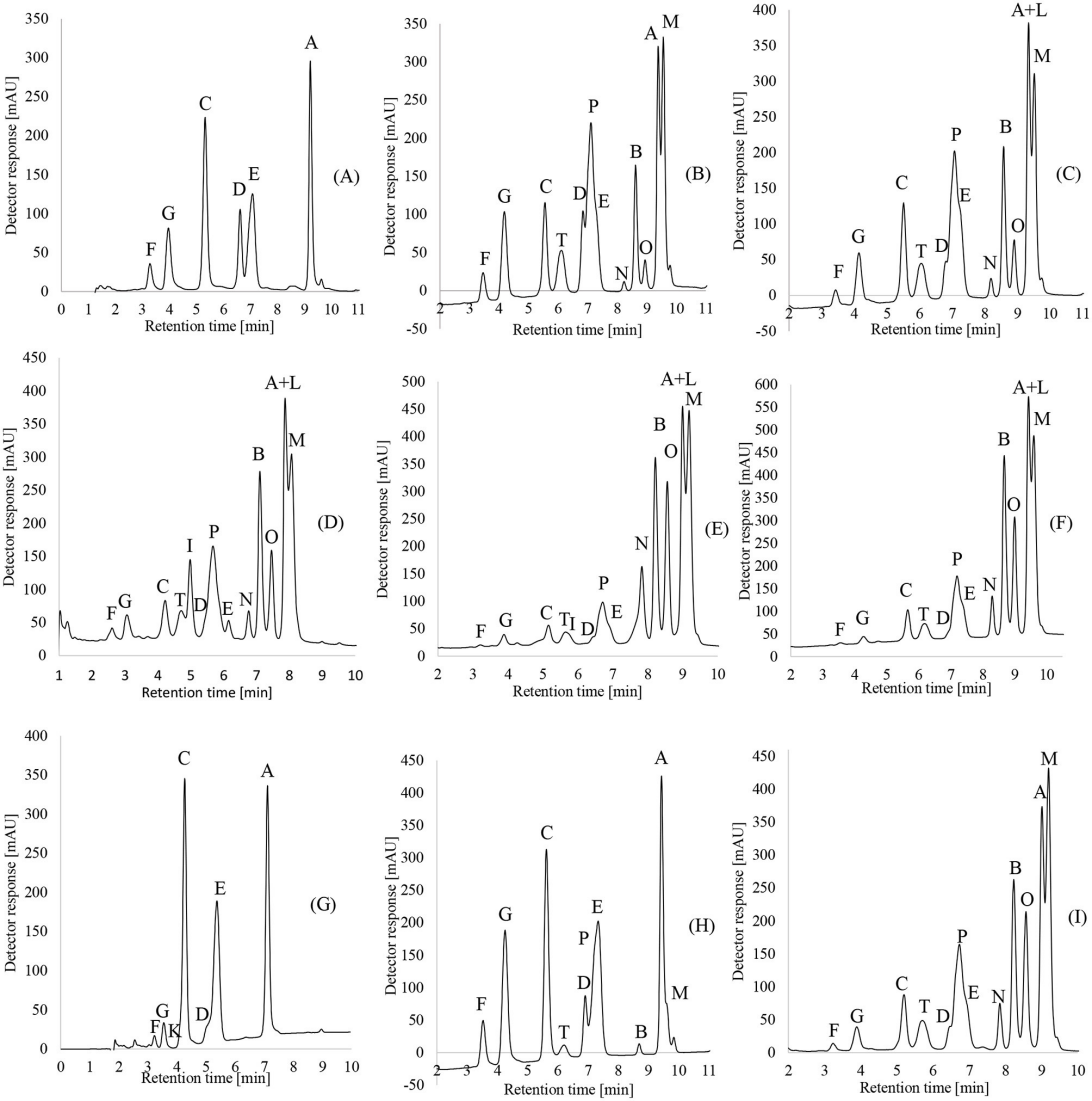
RP-HPLC detection and separations of CLPs from **(A)** “AN,” **(B)** “BL,” **(C)** “SP,” **(D)** “DM,” **(E)** “ES,” **(F)** “FA,” **(G)** “GA,” **(H)** “PB,” and **(I)** “VD” using a Kinetex^TM^ C18 2.6 μm column. Eluent settings were used: 0.5 ml/min flow rate; methanol and water isocratic elution as described in the “Methods” section. Liquid–liquid extraction was used as the extraction method.

Levels of oxidation of freshly analyzed flaxseed oil samples from “AN,” “BL,” “DM,” “ES,” “FA,” “PB,” “SP,” and “VD” were compared to the aged flaxseed oil sample from the “GA” manufacturer, produced in 2015. “GA” flaxseed oil was used as the reference for an oxidation pattern and oil with an unappealing content. All the above-mentioned manufacturers are commonly found on the shelves of grocery stores in Central Europe, especially Austria and Germany. The degree of oxidation of CLPs is considered to be a key element in the assessment of flaxseed oil quality (23, 24). Additionally, the bitterness of the flaxseed oil would depend mostly on the concentration of CLP-E in the oil (23, 24). There are many factors that affect the degree of oxidation of the oil, namely, the quality and level of oxidation of seeds, the methods used during flaxseed oil pressing and extraction, and the handling of the oil during packaging. The samples shown below are measurements of fresh flaxseed oil, aiming to avoid prolonged deterioration and oxidation (Figure 2). The pathways of CLP oxidation are shown in Table 3.

**Table 3:** Oxidative pathways of CLPs [according to Lao et al. (17)].

	**0 Met**		**1 Met**		**2 Met**	
0 Mso	CLP-A ILVPPFFLI	CLP-B MLIPPFFVI	CLP-L MLVFPLFI	CLP-M MLLPFFWI	CLP-N MLMPFFWV		CLP-O MLMPFFWI	
		↓	↓	↓	↙	↘	↙	↘
1 Mso		CLP-C **Mso**LIPPFFVI	CLP-E **Mso**LVFPLFI	CLP-D **Mso**LLPFFWI	CLP-T **Mso**LMPFFWV	CLP-I ML**Mso**PFFWV	CLP-H **Mso**LMPFFWI	CLP-P ML**Mso**PFFWI
					↘	↙	↘	↙
2 Mso		↓	↓		CLP-F **Mso**L**Mso**PFFWV		CLP-G **Mso**L**Mso**PFFWI	
1 Msn		CLP-K **Msn**LIPPFFVI	CLP-J **Msn**LVFPLFI			

*The oxidation of the methionine thiol results in the formation of sulfoxide (Mso) or sulfone (Msn)*.

The presence of methionine-containing peptides and the absence of methionine sulfoxide, as well as methionine sulfone-containing peptides, is a prerequisite for high-quality flaxseed oil. The complete absence of doubly oxidized CLP-F and CLP-G or their presence in low levels would be highly desirable and a good indication of high oil quality. The CLP patterns and their levels of oxidation in different flaxseed oil samples are shown in Figure 2.

**Figure 3:**
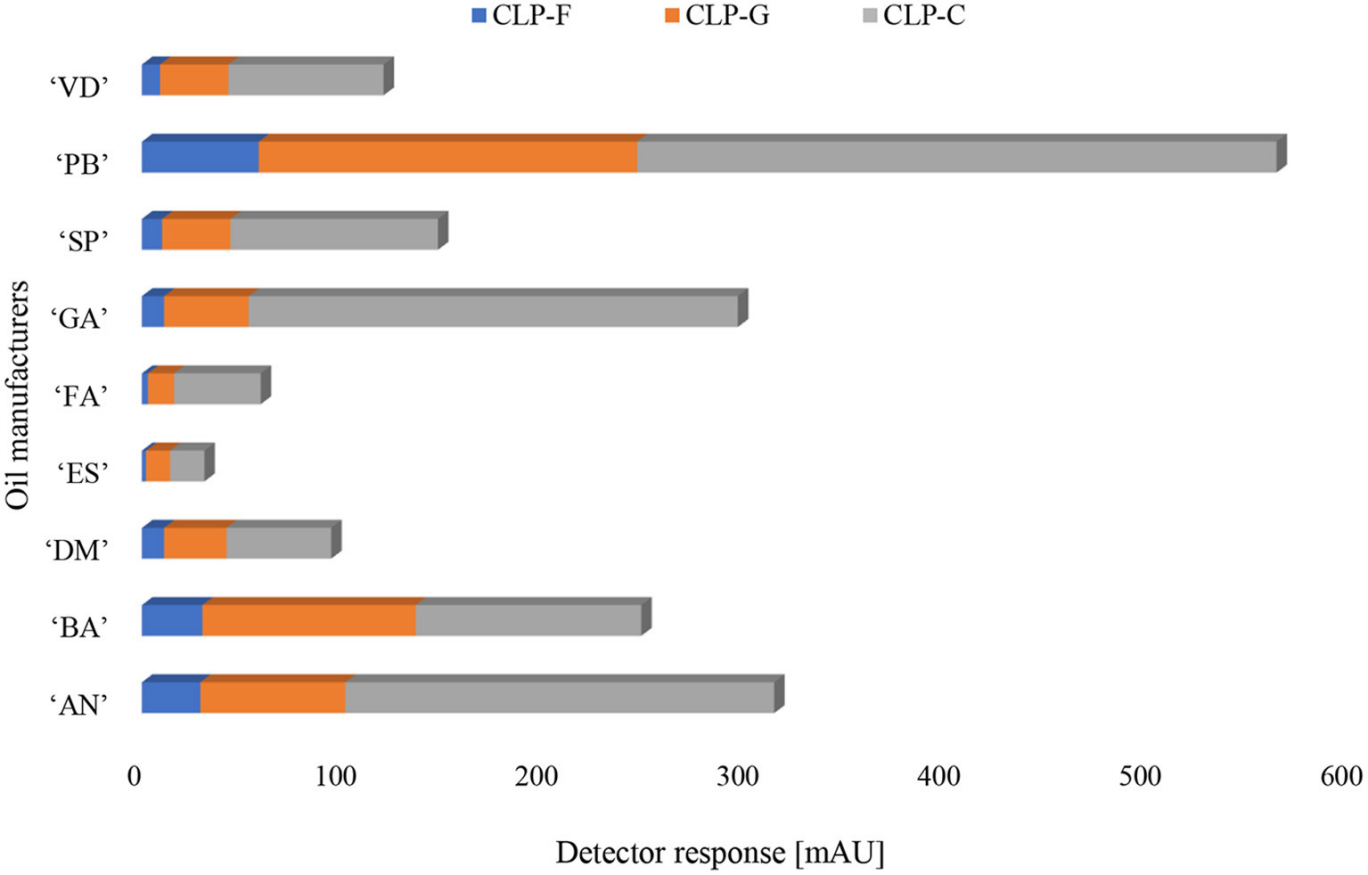
Comparison of CLP-C, CLP-F, and CLP-G, which occur in all samples.

The CLP patterns in flaxseed oil from nine different manufacturers showed that most oil samples have low levels of methionine sulfoxide, which is an indication of high oil quality. There was no detection of the peptides containing methionine sulfone, except for the “GA” sample from 2015, where CLP-K was detected, as would be expected for this aged sample. Additionally, CLP-E (indicator of bitterness) had the highest levels in the “GA” manufacturer, as it was forecasted in comparison to the other manufacturers. “AN” was the manufacturer with the second highest levels of oxidized CLP-E. Interestingly, doubly oxidized CLP-F and CLP-G had the highest levels in “PB.” The levels of CLP-E in this sample were the third highest among the manufacturers. “BL” manufacturer was flaxseed oil with the second highest levels of CLP-F and CLP-G. Among all the examined manufacturers, “ES,” “FA,” and “DM” samples had the highest levels of methionine-containing CLPs and the lowest levels of oxidized peptides CLP-F and CLP-G, as well as the lowest levels of “bitter peptide” CLP-E. Additionally, example data demonstrating important comparisons between all samples are shown in Figure 3. CLP-C, CLP-F, and CLP-G are compared in all samples.

### CLP Stability at Room Temperature and 90°C Over a Period of 60 Days

**Figure 4:**
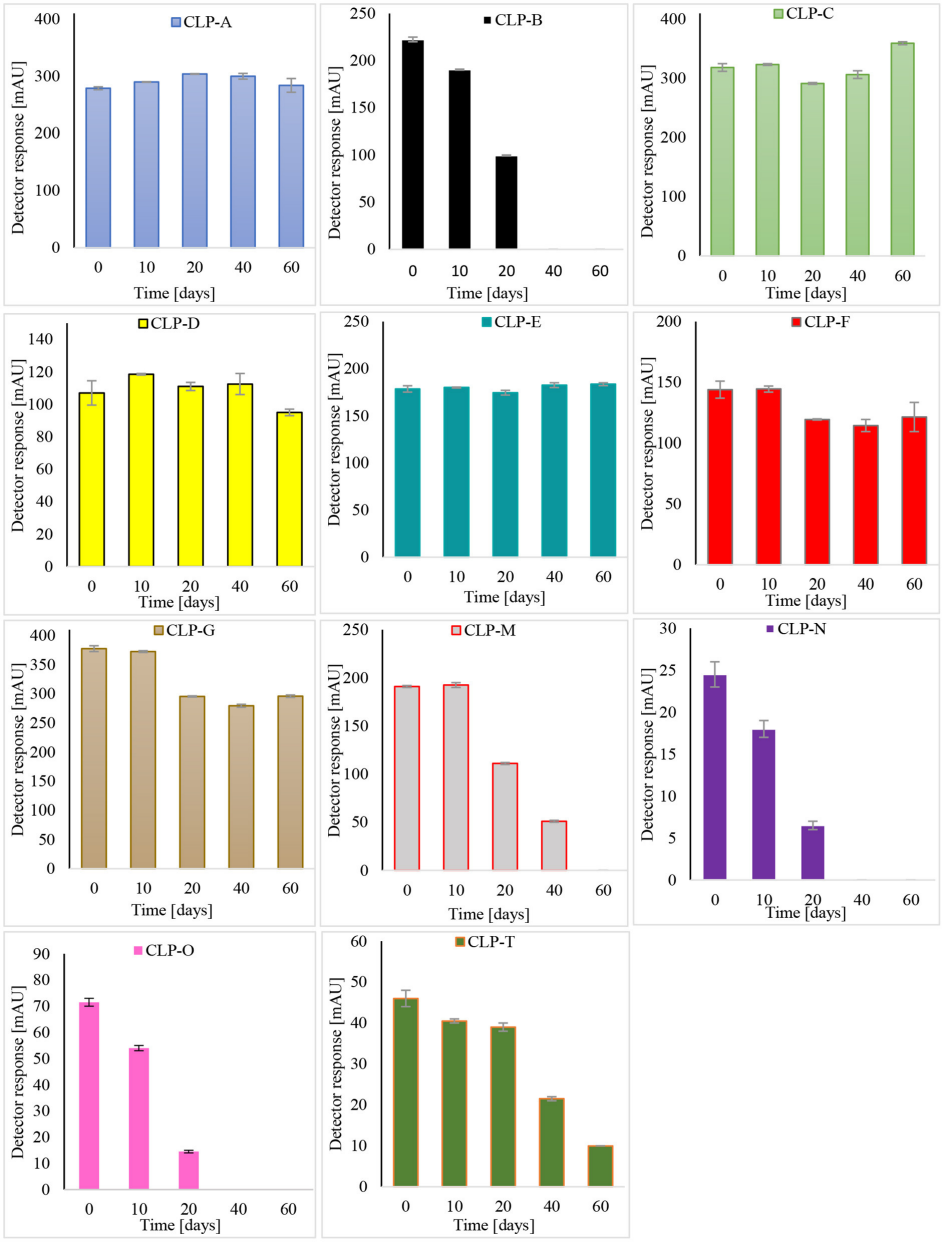
Stability of CLPs at room temperature.

The stability of 11 CLPs was examined over a period of 60 days in two flaxseed oil samples, namely, “PB” and “FA.” CLP-B, CLP-N, CLP-M, CLP-O, and CLP-T were observed in the “FA” sample, while the remaining CLPs were observed in the mild-aged aliquot of the “PB” flaxseed oil sample. The oxidation and behavior of CLPs were observed at room temperature (25°C) and 90°C. The stability of CLPs at room temperature is shown in Figure 4.

The least stable CLP at room temperature was CLP-O, where 80% of it degraded during the first 20 days, followed by CLP-N and CLP-B. All three CLPs were completely converted into their isomers, CLP-G, CLP-F, and CLP-C, within 40 days. CLP-M and CLP-T were also among the unstable CLPs. The levels of CLPs decreased by 45% and 80% over 40 days, whereas CLP-M was completely converted to CLP-D within 60 days. CLP-A, CLP-C, and CLP-E did not oxidize over a period of 60 days. Slight degradation of CLP-F and CLP-G was observed. It is important to mention here that in the case of the “FA” sample, CLP-B, CLP-N, CLP-M, CLP-O, and CLP-T would degrade over time, and peptides C, D, F, and G would increase accordingly. As the oxidation of peptides B, N, M, O, and T has already taken place in the case of the “PB” sample, this conversion would not be observed. Therefore, in the sample “PB,” the oxidation of peptides would not interfere with the examined temperature influence, and this oil is more suitable for the examination of the individual stability of the peptides C, D, F, and G. “FA” would be the sample of choice for the examination of the dynamics of CLPs at specific temperatures.

Interestingly, Aladedunye et al. observed almost constant CLP levels in flaxseed meals over extended periods of several months. A possible explanation for this could be the presence of an antioxidative system in flaxseed meals (24). Here, we report the complete evanescence of three CLPs within 40 days at 25°C.

**Figure 5:**
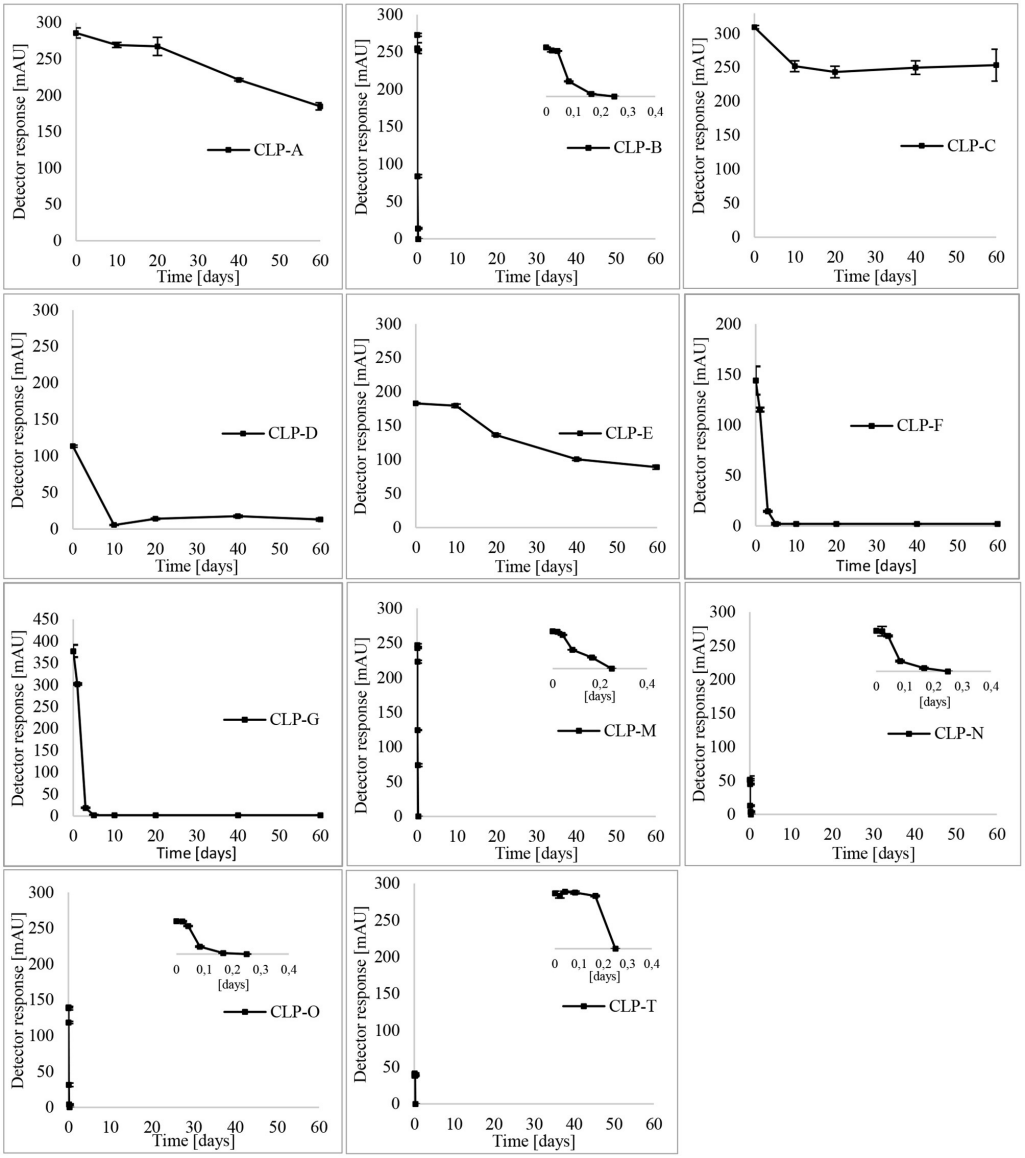
Degradation of 11 CLPs at 90°C from a mild-aged mixture of “PB” and fresh “FA” oil sample.

Significant changes were expected to occur at 90°C, even during the very first hours of heat exposure. Due to the accelerated oxidation during the very first hours, a different graphical representation compared to room temperature was used to observe changes in the very first hours of heat exposure (Figure 5).

After half an hour of heat exposure, CLP-O, CLP-B, and CLP-N showed identical degradation. However, after 2 h, CLP-O showed the highest rate of oxidation, where 80% of degradation was observed, following the degradation of CLP-N and CLP-B with 75% and 70% decreases in the levels of CLPs. The levels of CLP-M decreased by 50% after 2 h and by 67% after 4 h. The above-mentioned CLPs degraded after 6 h. CLP-T was stable for 4 h, with no indication of oxidation, and after 6 h, the CLPs were degraded completely. Even though the trends of CLP-F, CLP-G, and CLP-C stability shown here are from the “PB” sample, in the “FA” sample, these peptides would increase accordingly with the degradation of CLP-B, CLP-N, CLP-M, CLP-O, and CLP-T and reach their peak after 6 h of heating, after which the degradation phase starts. This increase in the concentration of primary oxidation products was only observed in “FA” and not in “PB,” as in “PB,” the oxidation process had already taken place. After 3 days, CLP-F decreased by 90% and remained constant at lower levels, while CLP-G was reduced by 95% and remained constant later. CLP-D was stable in the first several days. However, 5 days after heat treatment, the amount of CLP-D decreased by 90%. In a period of 5 days, CLP-C was reduced by 20%, CLP-E by 25%, and CLP-A by approximately 7%. Interestingly, after prolonged periods, CLP-C remained at reduced levels, while CLP-E and CLP-A deteriorated further. Over a period of 60 days, CLP-C was more stable than CLP-A. The order of the stability of the CLPs is as follows: O < N < B < T < M < G < F < D < E < C < A.

In a publication from 2013, CLP-N, CLP-O, CLP-B, and CLP-L were observed to be the fastest degrading peptides (24). They also reported CLP-A to be the most stable peptide, together with CLP-C and CLP-E (24). Overall, the group used lower temperatures, resulting in significantly lower oxidation rates compared to our results. However, a similar pattern for almost all CLPs was observed (24).

### Detection of Major CLPs in Flaxseed Oil Extracts Using High-Resolution MS

**Figure 6:**
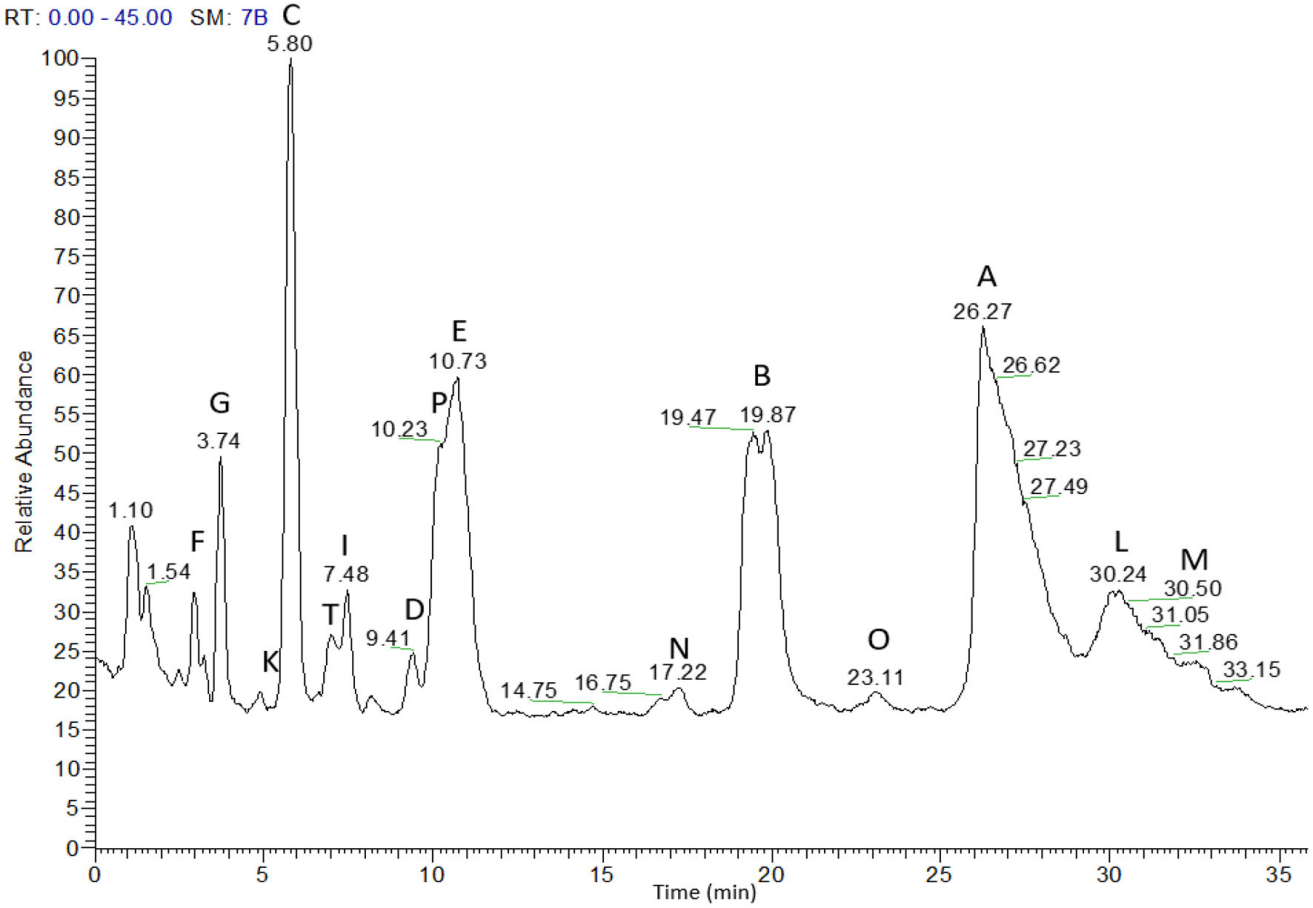
RP-HPLC-Orbitrap MS analysis of flaxseed oil extract using a Kinetex^TM^ C18 column.

Detection of the major CLPs in a mixture of moderately aged and fresh flaxseed oil extracts was examined using a Q Exactive Hybrid Quadrupole-Orbitrap Mass Spectrometer coupled with RP-HPLC (Figure 6).

In total, 15 cyclic peptides could be identified with elution orders F, G, K, C, T, I, D, P, E, N, B, O, A, L, and M. Aladedunye et al. (24) identified 14 CLPs in flaxseed oil, F, G, C, E, I, D, H, N, B, L, A, J, M, and K, using a Kinetex Column and HPLC Exactive Orbitrap MS. Lao and his group identified 15 CLPs, F, G, C, E, T, H, D, I, P, B, A, L, N, O, and M, using a Kinetex^TM^ phenyl-hexyl column and MALDI quadrupole/TOF prototype mass spectrometry (17). In 2021, in the CLP isolation experiment, 15 CLPs were identified using a Kinetex^TM^ phenyl hexyl column with a TripleTOF 5600 mass analyzer (32). Other groups identified up to 7 CLPs using Hybrid Quadrupole-TOF MS/MS with monolithic and microparticulate columns (29).

The phenomenon of peak splitting was previously reported in 2013 and 2014 for the peptides CLP-I and CLP-P (17, 24), while peak splitting for CLP-B was observed in this study. Usually, peak splitting was observed only in freshly prepared flaxseed oil on columns with higher separation efficiency (17).

## Conclusion

Complete separation of 12 CLPs was achieved using a fast and economical approach within just 10 min, using RP-HPLC with a standard Kinetex^TM^ C18 column and methanol/water as the eluent. Most of the CLPs ever separated using a methanol eluent are reported in this study. A Vydac^TM^ C18 5 μm column, which is an industrial standard for peptides and proteins, was examined for separation purposes. However, better separation was observed for Gemini^TM^ and Kinetex^TM^ columns. A novel method has been introduced, where the extraction of CLPs was significantly improved with the simple heating of a methanol–water mixture before liquid–liquid extraction. The presence of CLPs was confirmed using Hybrid Quadrupole Orbitrap Mass Spectrometer coupled with RP-HPLC, and a total of 15 CLPs could be identified. Levels of peptide oxidation, presence of the bitter compound “CLP-E,” and degree of oil deterioration would significantly vary in flaxseed oils commonly found on shelves in grocery markets. “GA,” ”AN,” and “PB” had the highest levels of CLP-E. Among all examined manufacturers, “ES,” “FA,” and “DM” samples had the highest levels of methionine-containing CLPs, the lowest levels of oxidized peptides, and lower levels of the “bitter” peptide CLP-E.

The pattern of CLP oxidation was examined at room temperature and 90°C, and the conversion of methionine-containing peptides into methionine sulfoxide-containing peptides was followed over a period of 60 days. At room temperature, the least stable CLP was CLP-O, where 80% of it degraded during the first 20 days, followed by the unstable CLP-N and CLP-B. All three peptides were completely converted into their isomers within 40 days. The same degradation pattern is observed at 90°C. However, with significantly faster oxidation, the abovementioned peptides were degraded in just 4 h. The order of stability of the CLPs is as follows: O < N < B < T < M < G < F < D < E < C < A.

## Data Availability Statement

The raw data supporting the conclusions of this article will be made available by the authors, without undue reservation.

## Author Contributions

AF conceived the main work on chromatography and stability experiments. H-JL performed the MS experiments. MM was responsible for the experimental design. All authors contributed to the writing of the manuscript. All authors contributed to the article and approved the submitted version.

## Conflict of Interest

The authors declare that the research was conducted in the absence of any commercial or financial relationships that could be construed as a potential conflict of interest.

## Publisher's Note

All claims expressed in this article are solely those of the authors and do not necessarily represent those of their affiliated organizations, or those of the publisher, the editors and the reviewers. Any product that may be evaluated in this article, or claim that may be made by its manufacturer, is not guaranteed or endorsed by the publisher.
